# Effect of rock loading rate based on crack extension and propagation

**DOI:** 10.1038/s41598-022-12759-4

**Published:** 2022-05-24

**Authors:** Mengze Yang, Houxu Huang, Yu Yang

**Affiliations:** 1 College of Civil Engineering, Liaoning Technical University, Fuxin, China; 2 Key Laboratory of Intelligent Underground Detection Technology, Anhui Jianzhu University, Hefei, China

**Keywords:** Engineering, Civil engineering

## Abstract

When subjected to constant static load, after a period of damage accumulation and crack development, the rock will fail under a load lower than its compressive strength. The transform of loading rate may have a certain influence on the mechanical properties of rock. In order to investigate the effect of loading rate on mechanical properties of red sandstone, the propagation form of internal cracks in the subcritical propagation stage in rock under static loading is defined as tensile. Based on Maxwell model, the expression of effective tensile stress for crack extension in rock is deduced, which explains the phenomenon of rock tensile failure. Based on the uniaxial compression test of red sandstone under different loading rates, and the surface deformation field of specimens is analysed with the method of digital image correlation, and the corresponding relationship between the loading rate effect and the change of mechanical properties as well as the energy accumulation and release characteristics is analysed, the phenomenon of rock tensile failure is further verified. This research can be effectively applied to geotechnical engineering disaster warning.

## Introduction

When subjected to constant load, after a period of damage accumulation and crack development, the brittle rock will often fail under a load lower than its compressive strength. Loading rate effect of brittle materials refers to the phenomenon that its mechanical properties are affected and changed with the change of loading rate^1–4^. The loading rate reflected in the actual project is the change of excavation rate. In mining engineering, tunnel engineering, water conservancy and hydropower engineering and other engineering practices, high-intensity excavation will lead to the emergence of loading rate effect, often resulting in the collapse of working face or surrounding rock, rockburst and other dynamic disasters, serious threat to engineering safety and personnel safety. Rock, as a complex in nature, has a large number of micro-cracks in its interior. The instability failure of brittle rock is closely related to the expansion and growth of cracks in rock. Exploring the mechanical properties of rock under different loading rates plays an important role for disaster warning in geotechnical engineering.

Based on laboratory tests, theoretical analysis and numerical simulation, experts and scholars at home and abroad have carried out a series of studies on the effect of rock loading rate. Zhang et al.^5^ summarized the dynamic test technology of rock under medium and high strain rates in the form of a review. Su et al.^6^ carried out uniaxial compression tests of marble under six strain rates using rock mechanics test system, and analysed the influence of strain rate changes on rock mechanical parameters, energy accumulation and release, and rock fracture form. Zhou et al.^7^ studied the uniaxial tensile strength characteristics of marble under different loading rates, analysed the energy consumption under different loading rates, and revealed the experimental characteristics and internal mechanism of loading rate effect of hard brittle rock. Martin^8^ concluded that the compressive strength of rock decreases with the decrease of loading rate through uniaxial compressive test of rock. Zhou et al.^9^ established a dynamic constitutive model of rock under dynamic cyclic load of medium and low strain rates, which can well simulate strain rate effect and damage effect of rock materials. Fuenkajorn et al.^10^ conducted uniaxial and triaxial compression tests on salt rock under different loading rates, and analyzed the variation rules of elastic parameters under different loading rates. Cui et al.^11^ investigated the effect of loading rate on the tensile strength and fracture surface topography of granite, basalt and limestone. According to the research of Alneasan et al.^12^, fracture toughness of brittle rock is an essential factor for analysing and designing many rock mechanics problems such as crack initiation and propagation, hydraulic fracturing and geothermal energy in rock mass, and this parameter is closely related to the value of strain rate. Li et al.^13^ studied the mechanical properties and deformation of coal samples under different strain rates. According to their studies, the effect of strain rate on the mechanical properties of coal samples mainly occurred in the peak stage and post-peak stage. The results show that the loading rate plays an important role in the study of rock mechanical properties.

Based on the theoretical relationship between microscopic crack growth and macroscopic deformation failure in brittle rocks, and from the angle of crack growth and propagation, this paper attempts to explain the mechanism of crack growth in brittle rocks under the effect of loading rate.

## Propagation characteristics of cracks in rocks

**Figure 1:**
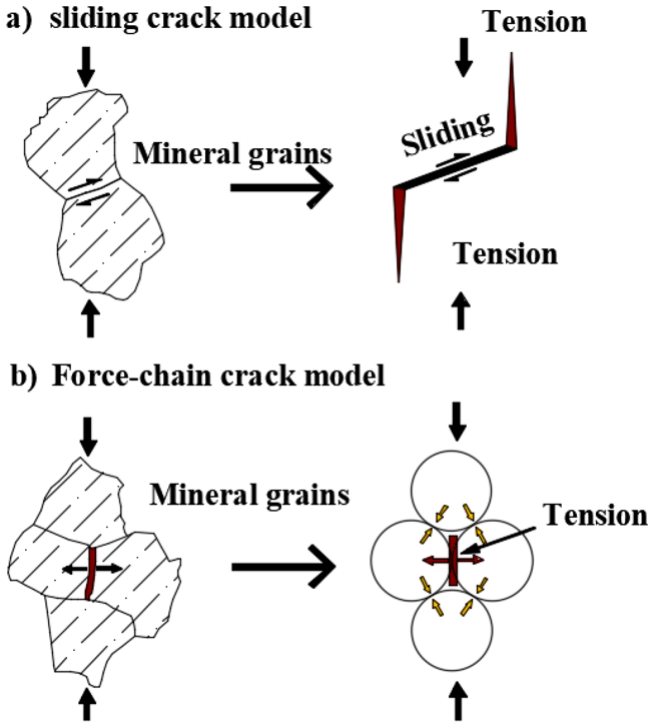
Two crack propagation modes in rocks^17^.

The propagation and growth of cracks in brittle rocks are the main factors leading to their damage, instability and failure^14–17^. By summarizing a large number of existing research results, Hoek and Martin^17^ pointed out that in the macroscopic continuous deformation stage, i.e., in the subcritical growth stage of the microscopic crack, it can be considered that the microscopic crack in the brittle rock mainly extends by the shear sliding mode and tensile failure modes, as shown in Fig. 1. However, regardless of the microcrack propagation pattern, the failure mode of the crack tip was tensile failure in this stage. According to the theory of Griffith^18^, when subjected to compressive stress, cracks in rock will expand along the direction of maximum principal stress under the action of load, while the cracks will expand perpendicular to the direction of minimum principal stress under the action of tensile stress.

**Figure 2:**
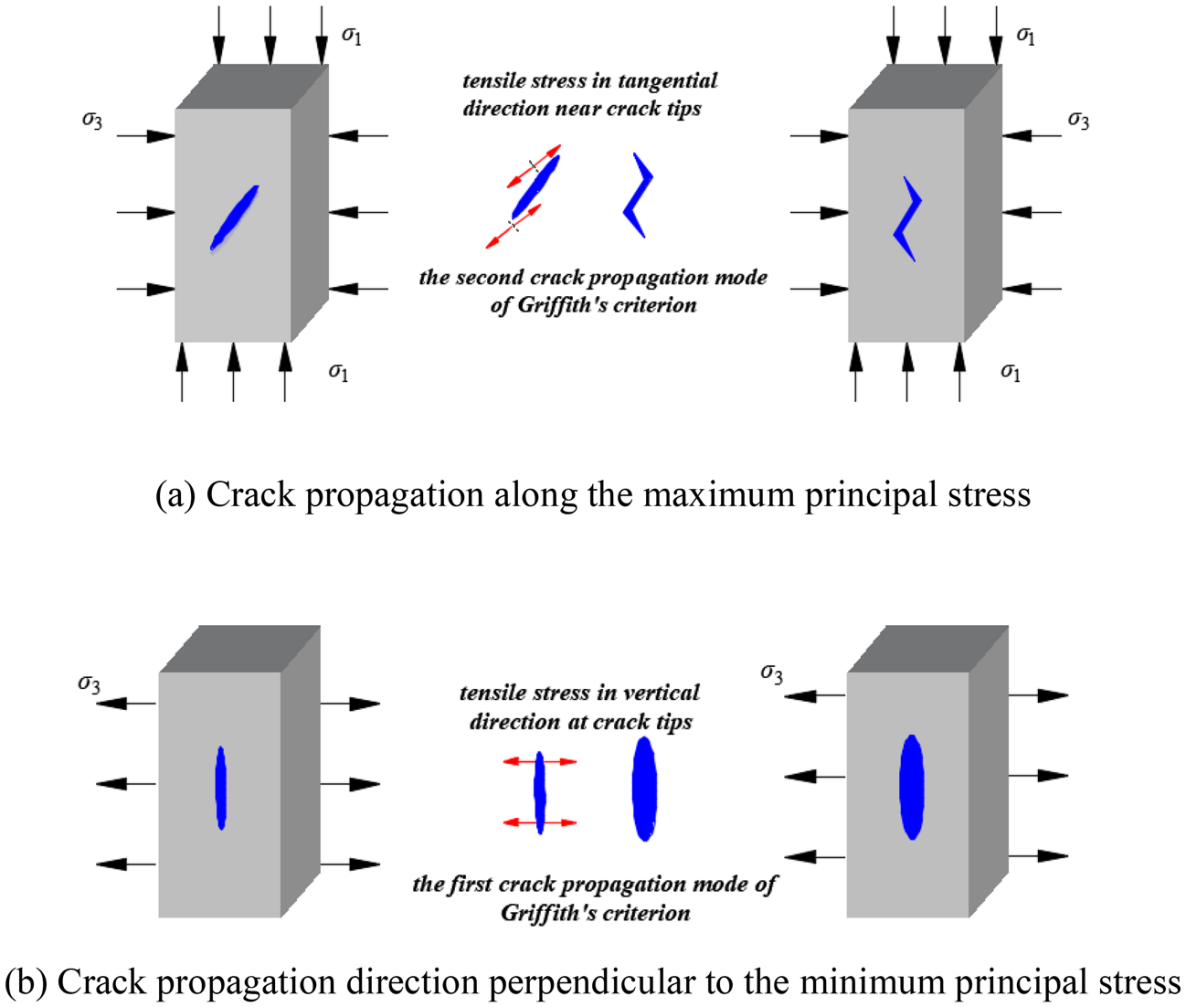
Two crack propagation modes^18^.

Applying Griffith theory to standard cuboid rock samples, no matter the rock samples are under compression or axial tension, no matter the cracks in the rock samples are shear slip type or tensile type, the growth direction of the rock samples will be approximately parallel to the axis of the cuboid rock samples, which are shown in Fig. 2. Based on the Griffith theory, the crack tip is extended in the form of tension, which can be equivalent to assume, the crack is affected by local tensile stress perpendicular to its growth direction in the local region near the crack tip. When applied to the cuboid specimen, the local tensile stress direction is perpendicular to the axis of the cuboid. That is to say, tensile stress perpendicular to the maximum principal stress $\sigma_{1}$ is generated in the compressed cuboid specimen.

## Tensile stress at crack tip

As a kind of brittle material, the inner structure of rock is very complex and heterogeneous^19,20^. Heterogeneity is the most typical characteristic of brittle rocks. Stress relaxation at the nonuniformities is the sole mechanism responsible for the dissipation of elastic energy in brittle rocks. The deformation and failure process of rock under compression is mainly composed of elastic deformation and brittle fracture, and the plastic deformation can be almost neglected. According to its deformation characteristics, Maxwell model can preferable describe the deformation and failure process of brittle rocks^21–23^.

In the author's previous studies, Griffith theory was applied to cylindrical rock column and Maxwell model was used to derive the expression of tensile stress induced by crack tip^24^. This paper will continue to apply this theory to cuboid rock specimens and deduce the expression of effective tensile stress for the extension of cracks in rock.

**Figure 3:**
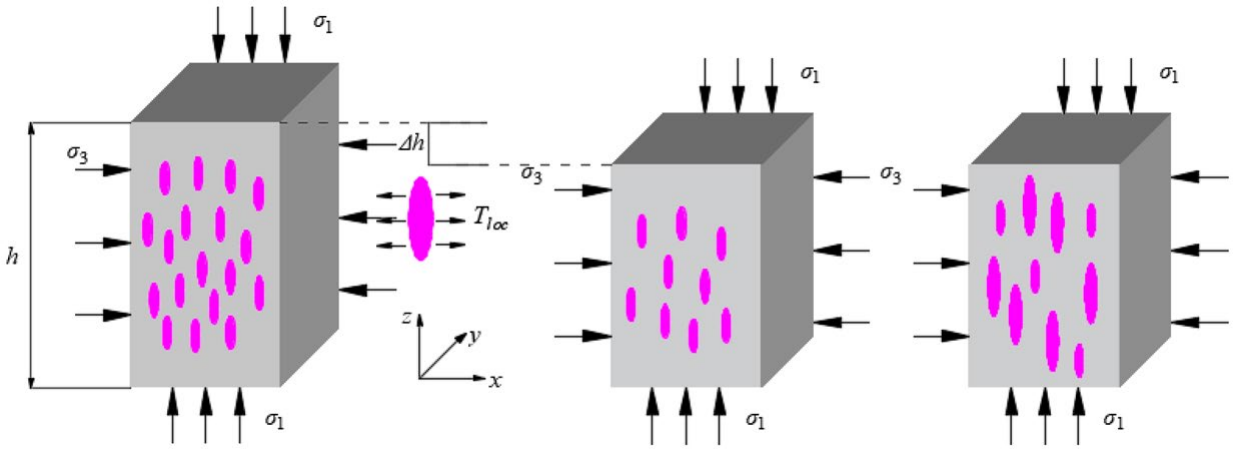
Local stress corresponding to loading progress.

Equivalent the brittle rock as a combination of elastic matrix and flat elliptic crack. In the loading process, the internal cracks of rock tend to expand along the direction of the maximum principal stress, so it is assumed that the long axis of the flat oval crack is parallel to the axial direction of the cuboid specimen. Figure 3 shows the expansion of the crack under the local stress during the loading process. The specific performance is as follows: the loading action increases the elastic stress in the elastic matrix of the rock, and the local tensile stress in the area around the crack is concentrated. Under the action of local tensile stress, the crack expands along the direction of the maximum principal stress, and the local tensile stress around the crack relaxes as the crack expands. So as to say, in the process of the rock deformation and failure, there exists two mechanisms of stress concentration and stress relaxation around the crack, and the tensile stress around the crack is acted on jointly by these two processes, which can be expressed by Maxwell model as follows^25^

1 $$ \frac{{d\Delta s_{ij}^{l} }}{dt} = 2\rho c_{s}^{2} \dot{e}_{ij} - \upsilon \frac{{\Delta s_{ij}^{l} }}{l} $$

where $\Delta s_{ij}^{l}$ represents the additional stress of the crack of scale *l*. $\dot{e}_{ij}$ denotes the deviation strain rate component corresponding to a given loading condition. $\rho$ represents the density of the rock. $\upsilon$ represents the relaxation rate of a single or multiple cracks. $c_{s}$ represents the shear elastic wave propagation velocity. ${l \mathord{\left/ {\vphantom {l \upsilon }} \right. \kern-\nulldelimiterspace} \upsilon }$ can be understood as the relaxation time required by the crack of size $l$ in the relaxation process. To simplify the analysis, it is assumed that the relaxation times of all additional stresses are uniform. The first term on the right of Eq. (1) represents elastic load, and the second term describes relaxation of additional stress during crack propagation.

By given a constant strain rate, the corresponding additional stress $\Delta s_{ij}^{l}$ can be expressed as

2 $$ \Delta s_{ij}^{l} = 2\rho c_{s}^{2} \dot{e}_{ij} \frac{l}{\upsilon }\left( {1 - e^{{\frac{ - \upsilon t}{l}}} } \right) $$

The initial point of rock instability failure in the process of loading is the occurrence of macroscopic fracture as a criterion, i.e. $t \gg \tau$^18^, Eq. (2) can be rewritten as

3 $$ \Delta s_{ij}^{l} \approx 2\rho c_{s}^{2} \dot{e}_{ij} \tau = 2\rho c_{s}^{2} \dot{e}_{ij} \frac{l}{\upsilon } $$

Defining the intensity of the additional stress as $\Delta \sigma_{ij} = \sqrt {{{3\Delta s_{ij}^{l} \Delta s_{ij}^{l} } \mathord{\left/ {\vphantom {{3\Delta s_{ij}^{l} \Delta s_{ij}^{l} } 2}} \right. \kern-\nulldelimiterspace} 2}}$, and substituting it into Eq. (3), we can obtain

4 $$ \Delta \sigma_{ij} \approx 3\rho c_{s}^{2} \dot{\varepsilon }_{ij} \frac{l}{\upsilon } $$

where $\dot{\varepsilon }_{ij} = \sqrt {{{2\dot{e}_{ij} \dot{e}_{ij} } \mathord{\left/ {\vphantom {{2\dot{e}_{ij} \dot{e}_{ij} } 3}} \right. \kern-\nulldelimiterspace} 3}}$ represents the strength of the strain rate.

Considering that $i = j = 3$, and according to $\dot{e}_{33} { = }\dot{e}_{3}$ and $\Delta \sigma_{33} { = }\Delta \sigma_{3}$, the local stress perpendicular to the loading direction generated in the process of the rock loading can be defined as

5 $$ \Delta \sigma_{3} = 3\rho c_{s}^{2} \dot{\varepsilon }_{3} \frac{l}{\upsilon } $$

The elastic–brittle plastic model is assumed to describe the mechanical behavior of the rock. Before reaching the uniaxial compressive strength, we assume that the deformation of the rock is elastic. At the same time, the compression and compression strains are defined as positive, while the tensile and tensile strains are defined as negative. Combining $E = 2\left( {1 + v} \right)\rho c_{s}^{2}$、$\varepsilon_{3} = - v\varepsilon_{1} = - v{{\sigma_{1} } \mathord{\left/ {\vphantom {{\sigma_{1} } E}} \right. \kern-\nulldelimiterspace} E}$, Eq. (5) can be rewritten as

6 $$ \Delta \sigma_{3} = 3\rho c_{s}^{2} \dot{\varepsilon }_{3} \frac{l}{\upsilon } = - \frac{3}{2}\frac{v}{1 + v}\sigma_{1} $$

According to Eq. (6), it can be found that tensile stress is generated in the direction perpendicular to the long axis of the crack. Ignoring the volume change of the specimen, the tensile stress $\Delta \sigma_{3}$ and its extreme value around the crack are respectively

7 $$ \Delta \sigma_{3} = - \frac{1}{2}\sigma_{1} $$

8 $$ \Delta \sigma_{3\max } = - \frac{1}{2}\sigma_{c} $$

Based on the research of Griffith, it can be found that $\sigma_{c} \approx 8\sigma_{t}$, where $\sigma_{c}$ and $\sigma_{t}$ represent compressive strength and tensile strength, respectively. Equation (8) can be rewritten as

9 $$ \Delta \sigma_{3\max } \approx 4\sigma_{t} $$

Equation (9) indicates that when the rock reaches uniaxial compressive strength, the maximum tensile stress around the crack in the rock is almost 4 times that of the tensile strength, which is enough to cause tensile failure along the axial direction perpendicular to the sample.

## Test of the rock loading rate effect

### Scheme of uniaxial-DIC test

The deformation failure of rock material is caused by the development of deformation localization in the rock system under most circumstances. At present, the analysis of rock deformation and failure process through the development of deformation localization has become one of the important methods to research rock mechanics, among which digital image correlation method has received extensive attention from scholars at home and abroad. DIC, the digital image correlation method, is a non-contact full-field nondestructive optical measurement method, which was first proposed in the 1980s^26–28^.

**Figure 4:**
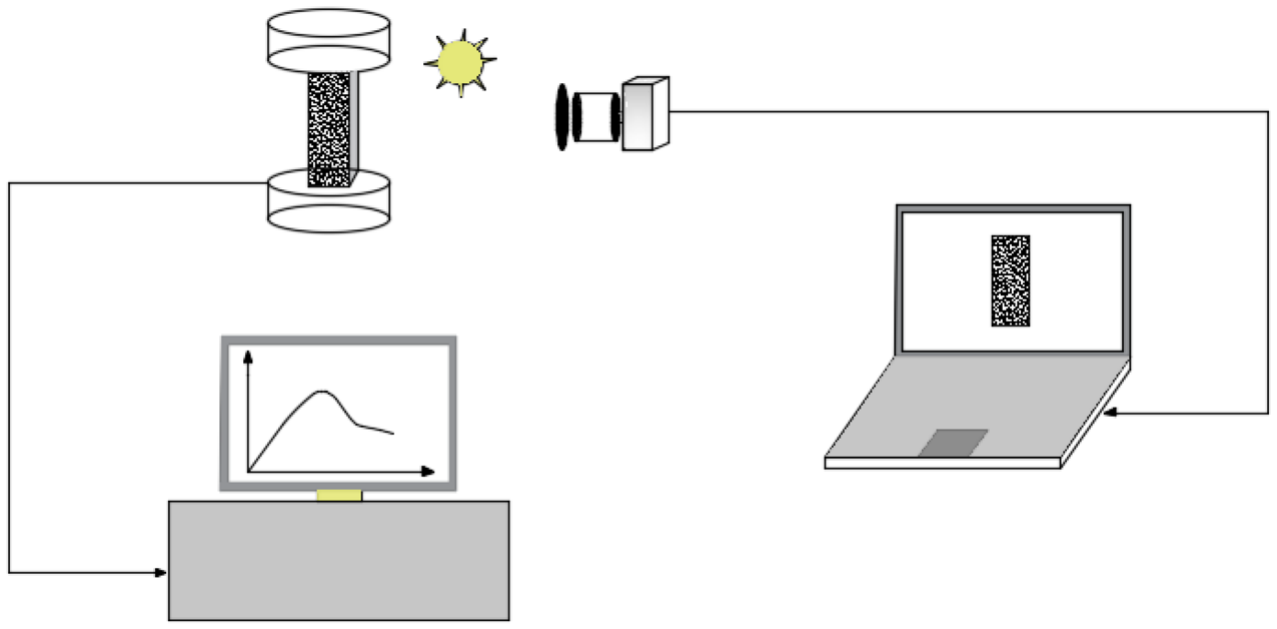
Uniaxial-DIC image of the testing system.

As shown in Fig. 4, uniaxial-DIC test is carried out to explore the loading rate effect of the rock, and the test system consisted of test loading system and data image acquisition system. The loading system is realized by hydraulic servo testing machine, and displacement control is used for axial loading. Selecting red sandstone for the sample and processed into the standard cuboid as $50\;{\text{mm}} \times 50\;{\text{mm}} \times 100\;{\text{mm}}$. The six end faces of the sample are polished to ensure that the end of the sample is smooth. Based on the relevant research^1,29–31^, the loading rates are selected for 0.02 mm/min, 0.1 mm/min and 0.5 mm/min, five groups are carried out at each loading rate, the test scheme is shown in Table 1.

**Table 1:** Uniaxial-DIC experiments and parameters.

Rock types	Hydrated	Loading rate	Number of sample
Red sandstone	Full of water	0.02 mm/min	Five samples were repeated at each loading rate
Red sandstone	Full of water	0.1 mm/min	Five samples were repeated at each loading rate
Red sandstone	Full of water	0.5 mm/min	Five samples were repeated at each loading rate

The specific operation steps of the test are as follows:

1. First of all, the white matte paint is sprayed on the surface of the sandstone specimen as the base color, and then the black matte paint is sprayed on the white background to form artificial speckles, which is randomly distributed, and the size of a single speckle is greater than three pixels. The ratio of the speckle area to the base color area on the surface of the specimen is close to 1:1.
2. Adjust the aperture and focal length of the camera to make the camera lens almost parallel to the surface of sandstone specimen.
3. The spray speckle specimens with good quality are placed on the test machine, and fixed under certain pressure.
4. Set the loading rate of the testing machine and the acquisition frequency of the camera.
5. Collect the DIC system image information, while the tester is pressurized.
6. When subjected to a certain pressure, obvious macroscopic damage occurs and the loading and image collection stop at the same time. Select the appropriate patch on the sandstone specimen to process and analyse the image, and obtain the deformation field of the sandstone specimen surface.

### Analysis of rock uniaxial-DIC test results

**Figure 5:**
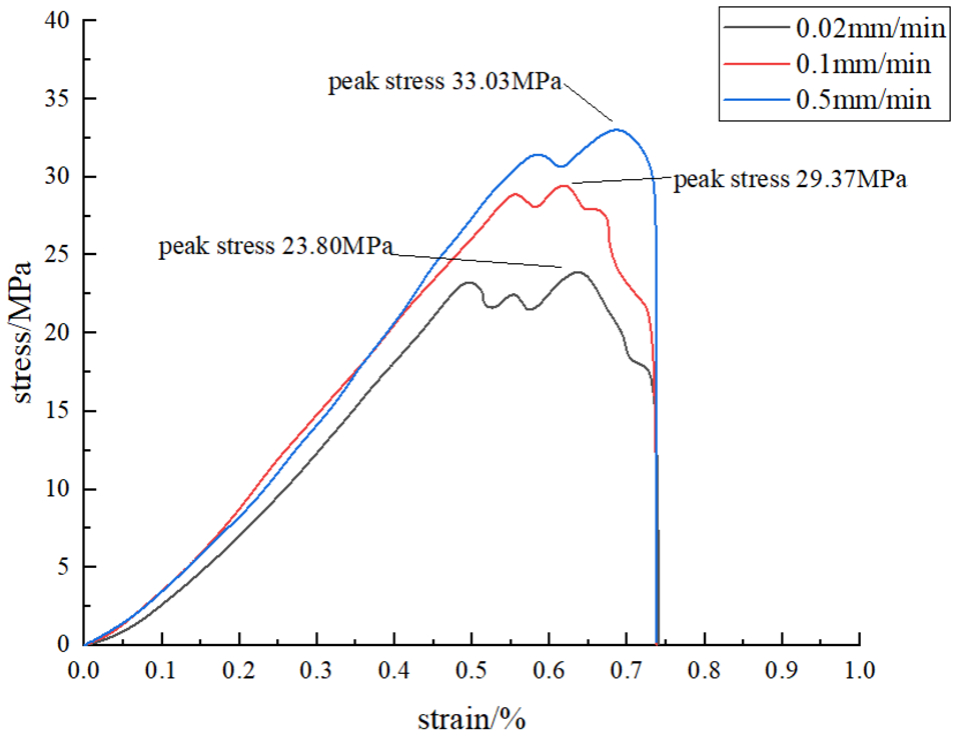
Loading curves at different loading rates.

Five groups of tests are carried out at each loading rate level. Seleting a group of data with better test results under each group of loading rates. The stress–strain curve of sandstone specimen during loading is shown in Fig. 5, and the test results are shown in Table 2.

**Table 2:** Test results in different strain rates under uniaxial compression.

Sample number	Loading rate (mm/min)	Peak stress (MPa)	Time required to reach peak stress (s)	Stress growth rate (MPa/s)	Modulus of elasticity (MPa)
Z11	0.02	21.5	1755.46	0.0122	4025.19
Z12	0.02	22.7	1810.25	0.0125	4086.32
Z13	0.02	23.8	1874.88	0.0126	4103.31
Z14	0.02	24.2	1924.33	0.0126	4156.48
Z15	0.02	24.8	1988.43	0.0125	4195.74
Z21	0.1	29.12	320.47	0.0909	4695.43
Z22	0.1	29.37	300.24	0.0978	4876.75
Z23	0.1	29.68	330.78	0.0897	4826.86
Z24	0.1	30.03	350.14	0.0858	4795.26
Z25	0.1	30.46	307.24	0.0991	4775.69
Z31	0.5	32.75	65.47	0.5002	4850.14
Z32	0.5	33.03	70.56	0.4681	4890.71
Z33	0.5	34.76	75.89	0.4580	4887.52
Z34	0.5	35.63	80.94	0.4402	4875.36
Z35	0.5	35.83	78.35	0.4573	4898.25

**Figure 6:**
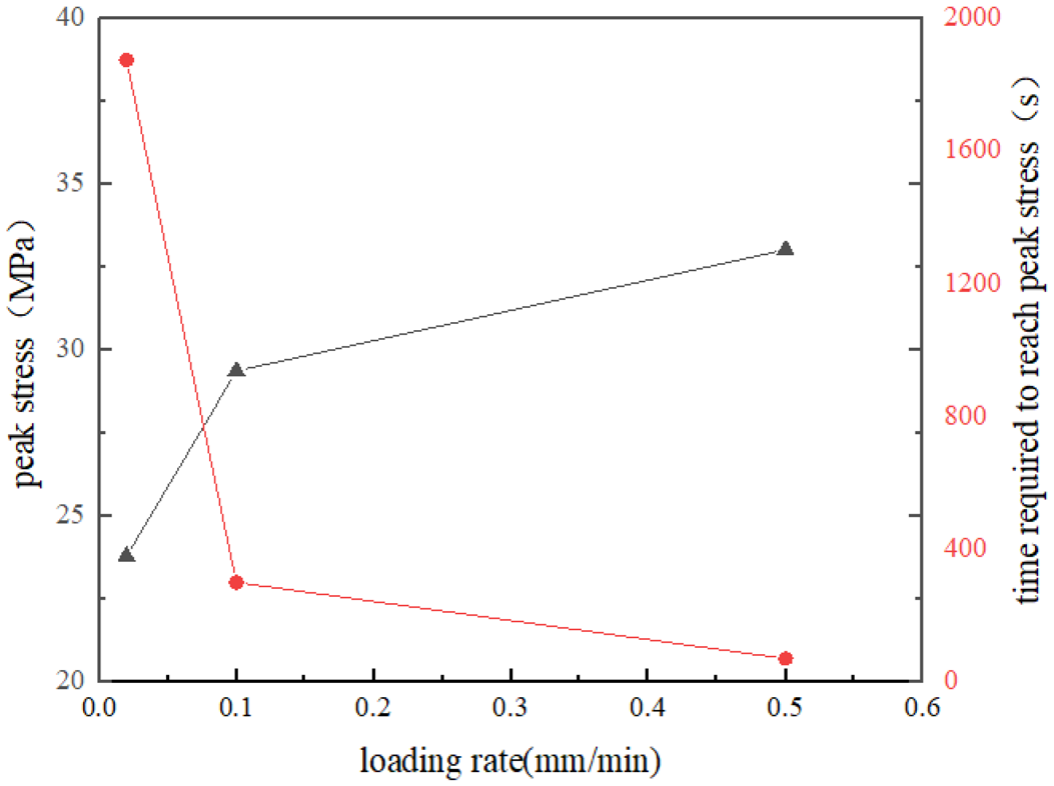
The relationship between peak stress and loading rate.

**Figure 7:**
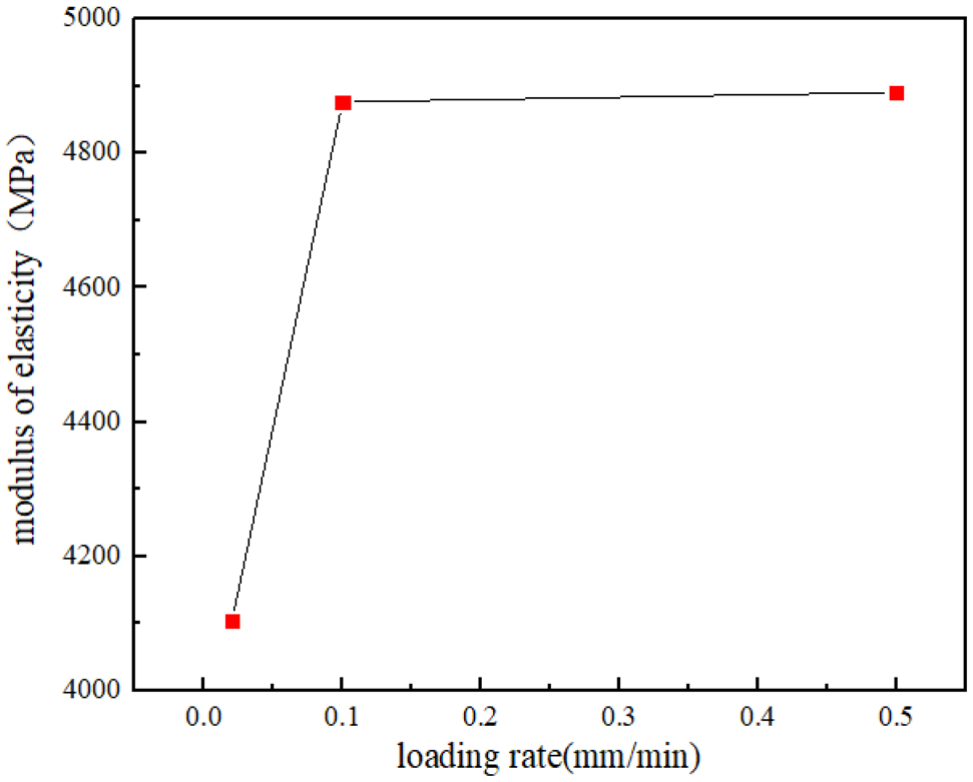
The relationship between modulus of elasticity and loading rate.

**Figure 8:**
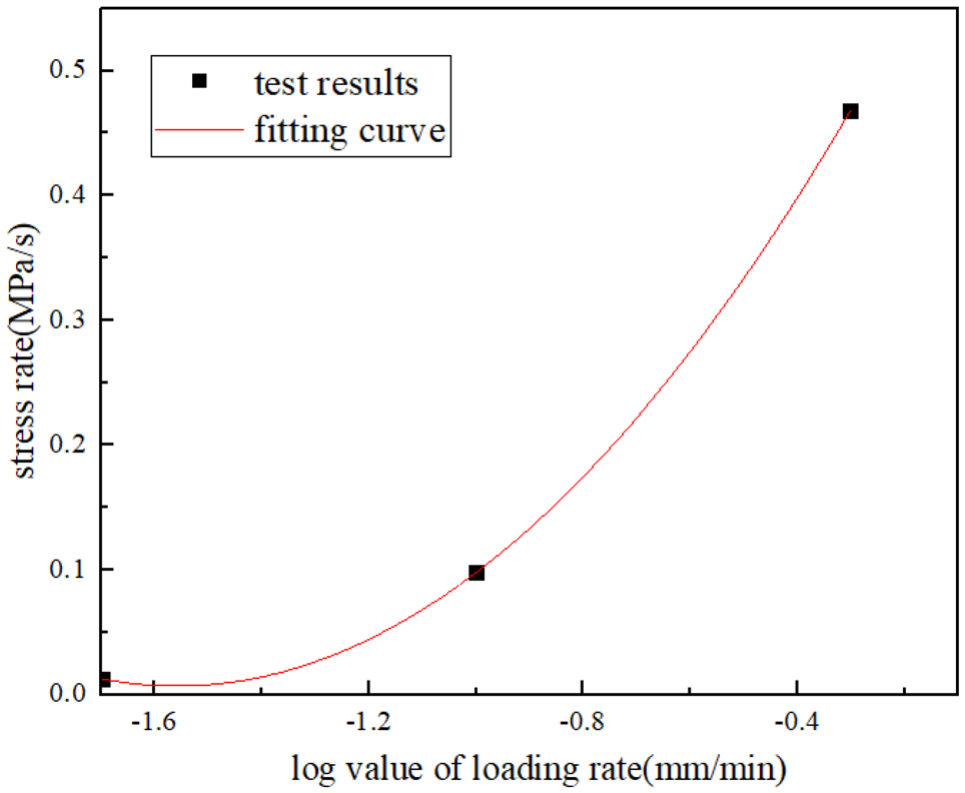
The relationship between stress rate and loading rate.

According to the stress–strain curve of sandstone and the test results, the peak strength and elastic modulus of sandstone increase with the increase of loading rate, and the time required for sandstone specimen to reach the peak stress gradually decreases. Figures 6, 7 and 8 show the relationship between loading rate and peak stress, time required to reach peak stress, modulus of elasticity and stress rate, respectively. As we can see, the peak stress increases with the increase of loading rate, which are positively correlated, while the time required to reach peak stress shows the opposite variation. The stress rate increases with the increase of the logarithm value of the loading rate. Through fitting, it is found that the two are non-linear and positively correlated. When the loading rate is small, the stress rate increases relatively slowly.

**Figure 9:**
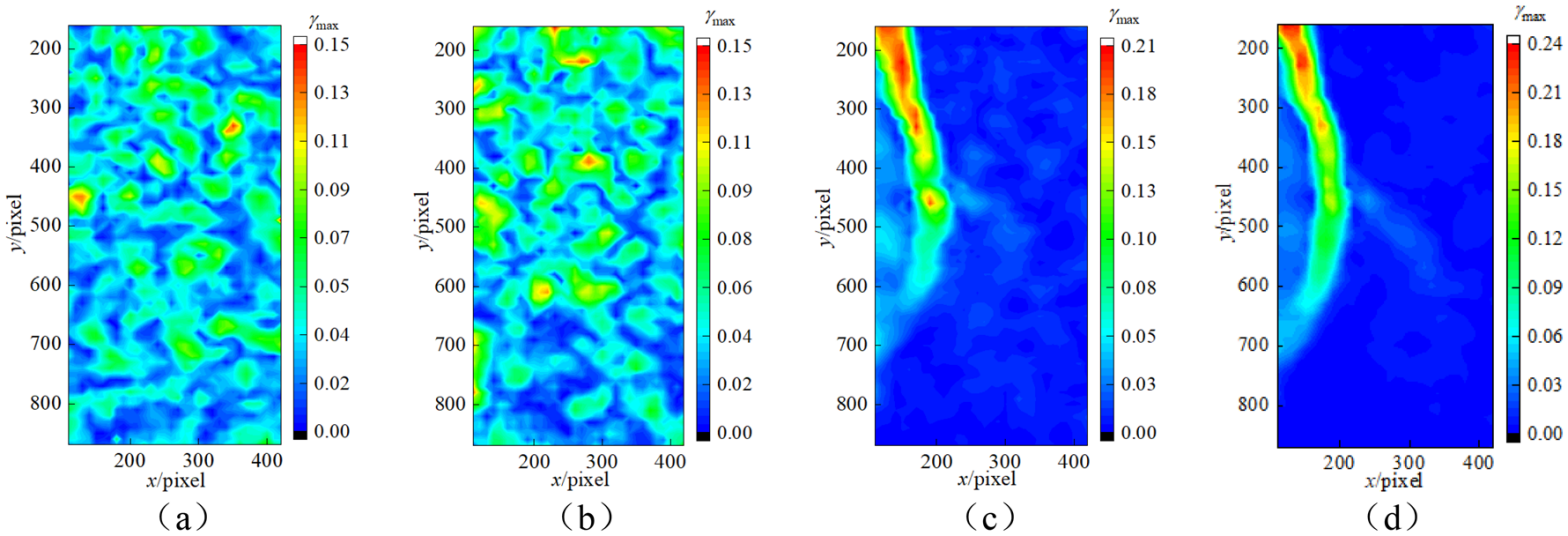
Nephograms of deformation field evolution (0.1 mm/min).

Taking 0.1 mm/min as an example to analyse the strain evolution characteristics of sandstone during loading. As shown in Fig. 9, selecting the speckle images on the surface of rock specimens at the beginning of loading as reference images. Based on the digital speckle correlation method to analyse the speckle images in the loading process, and then obtained the strain field evolution cloud map of sandstone.

At the initial stage of loading, the internal microcracks in sandstone are closed under pressure from the opening state in the initial state, resulting in large strain in local areas and random distribution of strain field. According to the analysis in the previous chapter, the deformation of rock before reaching compressive strength contains elastic deformation, and strain localization region begins to appear near the peak strength. The phenomenon of strain localization is the phenomenon of strain concentration in a small area of compressed sandstone before macroscopic failure. The area is narrow and developing continuously, and the failure of rock can be predicted accordingly. When the sandstone enters the strain-softening stage, the strain localization region gradually becomes narrower, and then the strain localization shear zone is formed in the strain localization region and runs through the whole sandstone specimen, followed by the macroscopic failure region of sandstone. After reaching the peak strength, with the decrease of stress, the microcracks in sandstone continue to expand. In the process of sandstone deformation and failure, elastic energy is released, and the released elastic energy maintains the further expansion of cracks. As the sandstone enters the residual strength stage, the cracks shear slip along the macroscopic fracture plane. At this time, the strain concentration occurs in the strain-localized shear band, while the strain field is evenly distributed outside the localized shear band and the deformation is relatively uniform. At this time, the sandstone is still in the elastic deformation stage. The location of localized shear zone, i.e., the final macroscopic fracture surface. The above phenomena can be verified by the strain field evolution cloud map.

When the sandstone specimen obtains in the natural state and subjected to non-load, the energy in the rock is relatively dispersed, and the energy field is approximately evenly distributed. Meanwhile, considering the heterogeneity and internal friction characteristics of rock, the internal energy of rock under compression will preferentially accumulate in the area with weak mechanical properties, resulting in uneven distribution of energy field. Before reaching the peak strength, energy is continuously input into the rock system through axial loading. Most of the energy is accumulated in the form of elastic energy, and only a small part of energy is used in the form of dissipative energy for the closure of the original micro-cracks in the rock and the formation of new cracks at the yield stage. As shown in Fig. 9, by combining with the strain evolution cloud diagram of sandstone at different times under the loading of 0.1 mm/min, it can be found that the deformation localization zone of rock begins to incubate from the left side of the top of the specimen, and the deformation localization zone is approximately parallel to the axis of the specimen. Before reaching the peak strength, there is no obvious macroscopic penetrating crack on the rock surface. When the peak strength is exceeded, as the load continues to increase, energy transfer to the area below and accumulation, leading to micro cracks on the surface of the specimen gradually expanded. When energy reaches the energy storage limit of rock, macro cracks are formed and eventually run through the whole specimen, resulting in the overall failure of rock. The macro failure of specimen is accompanied by the release of energy. The accumulation and release of energy is the essence of the destruction of rocks and other materials. According to the analysis, more energy is accumulated in the pre-peak stage, while in the post-peak stage, more energy is released, which drives the coalescence of cracks in the rock and leads to the instability failure of rock materials.

**Figure 10:**
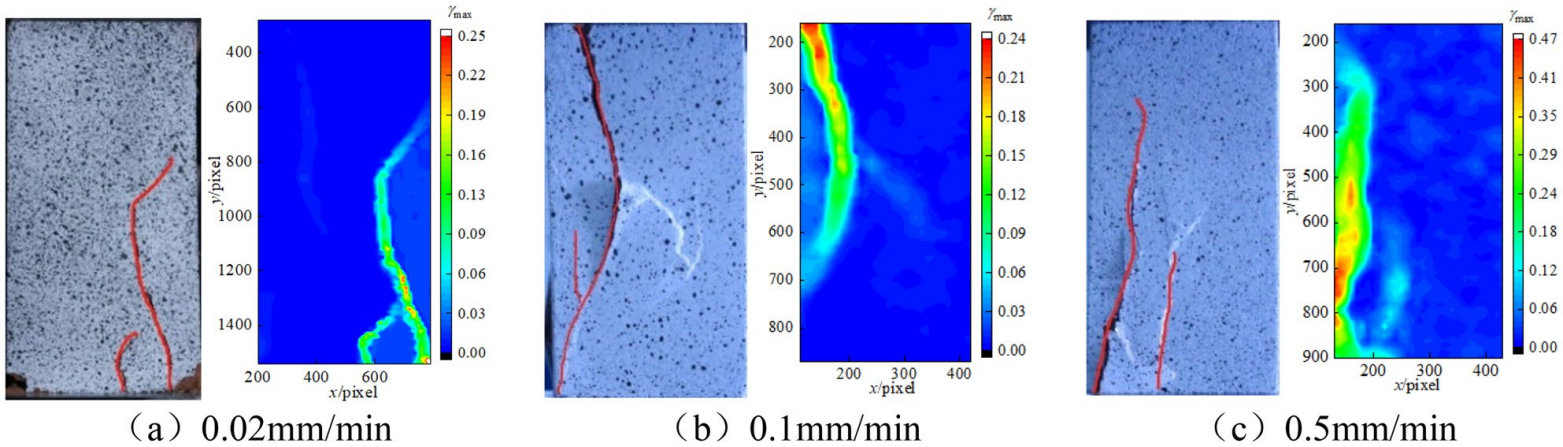
Peak deformation field and final failure form of specimens under three loading rates.

Due to the limitation of space, the paper only lists the comparison between the strain cloud at the peak point of rock and the real failure state, as shown in Fig. 10. The true failure state of rock is shown on the left, the surface crack of rock specimen is marked in red line, and the strain field evolution cloud is shown on the right. The final failure of sandstone under three different loading rates is composed of macroscopic fractures and multiple micro-fractures. In addition, under the action of different loading rates, the extension mechanical properties of the tip of the main fracture are mainly tensile.

## Conclusion

This paper takes cuboid sandstone under uniaxial load as the research object and theoretically deduces the expression of effective tensile stress leading to crack extension. Based on uniaxial-DIC test, the influence of loading rate on rock mechanical properties is analyzed, and the following conclusions are drawn:

1. Based on Maxwell equation, the expression of effective tensile stress for crack propagation in rock is derived. When the rock sample reaches uniaxial compressive strength, the induced tensile stress of the crack is close to 4 times of the tensile strength, which is enough to cause tensile failure of the rock sample along the direction perpendicular to the axis, thus explaining the phenomenon that the stress on the crack tip is still tensile stress even under compression.
2. When subjected to uniaxial load, the cracks first initiated at the upper or lower end faces of the specimen. With the continuous action of load, the cracks expanded and developed to the opposite side approximately parallel to the axis of the specimen, and gradually formed strain localized shear bands. Before the peak strength, no macroscopic transmissibility cracks appeared on the surface of the specimen. After reaching the peak strength, macroscopic penetrating cracks appear on the surface of the specimen under the action of loading, followed by macroscopic failure of the specimen, accompanied by a large amount of energy release.
3. The influence of loading rate effect on rock mechanical properties are as follows: with the increase of loading rate, the peak strength and elastic modulus and stress rate of rock increase, and the brittleness of rock becomes stronger.
4. The uniaxial-DIC test verifies the nature of rock failure, namely, the accumulation and release of energy. The energy accumulation in the pre-peak stage and the release of energy in the post-peak stage drive the growth and coalescence of cracks in the rock, thus leading to the instability and failure of rock materials.

## Data Availability

The datasets used and/or analysed during the current study available from the corresponding author on reasonable request.

## List of symbols

$\Delta s_{ij}^{l}$: Local additional stress component
$l$: Crack scale/crack length
$$\dot{e}_{ij}$$: Strain rate
$$\rho$$: Density
$\upsilon$: Rate of stress relaxation
$c_{s}$: Elastic wave velocity
$$\tau$$: Relaxation time
$$t$$: Loading time
$\Delta \sigma_{ij}$: The intensity of the additional stress
$\dot{\varepsilon }_{ij}$: Strength of the strain rate
$$\nu$$: Poisson’s ratio
$$E$$: Elastic modulus
$\sigma_{c}$: Uniaxial compression stress
$$\sigma_{t}$$: Tensile stress
